# Telomere Length and Long-Term Endurance Exercise: Does Exercise Training Affect Biological Age? A Pilot Study

**DOI:** 10.1371/journal.pone.0052769

**Published:** 2012-12-26

**Authors:** Ida Beate Ø. Østhus, Antonella Sgura, Francesco Berardinelli, Ingvild Vatten Alsnes, Eivind Brønstad, Tommy Rehn, Per Kristian Støbakk, Håvard Hatle, Ulrik Wisløff, Javaid Nauman

**Affiliations:** 1 K. G. Jebsen Center of Exercise in Medicine at Department of Circulation and Medical Imaging, Faculty of Medicine, Norwegian University of Science and Technology, Trondheim, Norway; 2 Department of Biology, University of Rome “Roma Tre”, Rome, Italy; 3 Department of Public Health and General Practice, Faculty of Medicine, Norwegian University of Science and Technology, Trondheim, Norway; 4 Centre for Sports and Physical Activity Research, Norwegian University of Science and Technology, Trondheim, Norway; Universidad Europea de Madrid, Spain

## Abstract

**Background:**

Telomeres are potential markers of mitotic cellular age and are associated with physical ageing process. Long-term endurance training and higher aerobic exercise capacity (VO_2max_) are associated with improved survival, and dynamic effects of exercise are evident with ageing. However, the association of telomere length with exercise training and VO_2max_ has so far been inconsistent. Our aim was to assess whether muscle telomere length is associated with endurance exercise training and VO_2max_ in younger and older people.

**Methods:**

Twenty men; 10 young (22–27 years) and 10 old (66–77 years), were studied in this cross-sectional study. Five out of 10 young adults and 5 out of 10 older were endurance athletes, while other halves were exercising at a medium level of activity. Mean telomere length was measured as telomere/single copy gene-ratio (T/S-ratio) using quantitative real time polymerase chain reaction. VO_2max_ was measured directly running on a treadmill.

**Results:**

Older endurance trained athletes had longer telomere length compared with older people with medium activity levels (T/S ratio 1.12±0.1 vs. 0.92±0.2, p = 0.04). Telomere length of young endurance trained athletes was not different than young non-athletes (1.47±0.2 vs. 1.33±0.1, p = 0.12). Overall, there was a positive association between T/S ratio and VO_2max_ (r = 0.70, p = 0.001). Among endurance trained athletes, we found a strong correlation between VO_2max_ and T/S ratio (r = 0.78, p = 0.02). However, corresponding association among non-athlete participants was relatively weak (r = 0.58, p = 0.09).

**Conclusion:**

Our data suggest that VO_2max_ is positively associated with telomere length, and we found that long-term endurance exercise training may provide a protective effect on muscle telomere length in older people.

## Introduction

Telomeres are nucleoproteic complexes located at the end of eukaryotic chromosomes composed by non-coding, repetitive (TTAGGG)-DNA sequences and a multitude of telomere-associated proteins (*e.g.,* TRF1 and TRF2) [1], [2]. As cells proliferate, TTAGGG repeats are lost from telomeres owing to the so called end-replication problem. Telomeres are hypothesized to function as mitotic clock by getting progressively shorter with every cell cycle, leading to erosion and dysfunction at cellular level, and are associated with cell cycle delay, triggering of DNA damage response, and apoptosis [1]. Moreover telomere erosion and/or dysfunction has been associated with several pathologies [3], [4], and recent reports have shown an association between shortened telomere length and increased risk of age-related outcomes [5], [6].

On the other hand, long-term exercise and higher aerobic cardiovascular fitness (VO_2max_) are associated with good health and improved survival [7], [8], and dynamic effects of exercise are evident with ageing [9], [10]. However, the inter-relation of exercise training and cardiovascular fitness with telomere length has so far been inconsistent, as few studies reporting a protective effect [9], [11]–[13] of exercise on telomere length, while others suggesting no such association [14], [15]. Therefore, we assessed whether muscle telomere length is associated with endurance exercise training, and evaluated the relationship between VO_2max_ and telomere length.

## Methods

### Study Population

We studied groups of young (n = 10, 22–27 years) and older (n = 10, 66–77 years) men. Five out of 10 young adults and 5 out of 10 older were endurance athletes, while other halves were exercising at a medium level of activity (non-athletes). We invited the participants who were residing within Trøndelag County in Norway. The older athletes were selected from the 58-km long Norwegian “Birkebeiner” cross country ski race [16], [17]. Five out of eight invited older athletes accepted to participate and fulfilled the inclusion criteria, i.e., >65 years of age at the time of participation in 2008 “Birkebeiner” cross country ski race, and had been actively training and participating in other ski competitions or “Birkebeiner” race in previous years, and healthy at the time of screening. Young athlete group (20–30 years of age) was selected on the basis of participation in “Birkebeiner” ski race together with other track running competitions. Age matched non-athlete control groups were selected if they had never participated or competed at higher levels in any sports, but were physically active; senior’s soccer and dance for at least two times a week was the most common in older population (n = 5) and exercising at medium intensities (breaking into sweat and losing the breath during exercise) for at least two times a week in young people (n = 5). The study was accomplished according to the Declaration of Helsinki and approved by the regional committee for medical research. Written informed consent was obtained from each participant.

### Measurement of Relative Telomere Length (T/S Ratio)

Muscle biopsies of the vastus lateralis were taken using a sterile 5 mm diameter biopsy needle (Pelomi, Denmark). Xylocain was used as local anesthetic and infiltrated beyond the depth of the biopsy several minutes before the procedure. Muscle biopsies were snap- frozen in liquid nitrogen immediately after sampling.

Genomic DNA was extracted directly from tissue samples using the SIGMA-ALDRICH GenElute Mammalian Genomic DNA Miniprep Kit, and relative telomere length was assessed using multiplex quantitative polymerase chain reaction [18]. The average relative telomere length was calculated as telomere repeat copy number/single-gene copy number (T/S), where T is the number of nanograms of the standard DNA that matches the experimental sample for copy number of the telomere template, and S is the number of nanograms of the standard DNA that matches the experimental sample for copy number of the single copy gene [18].

### Exercise Testing

An individualized protocol [19] was applied to measure maximal oxygen uptake. Each test-subject was familiarized with treadmill walking during the warm-up of 8–10 minutes, also to ensure safety and avoid handrail grasp when this was not absolutely necessary. Oxygen uptake kinetics were measured directly by a portable mixing chamber gas-analyzer (MetaMax II, Cortex, Leipzig, Germany) with the participants wearing a tight face mask (Hans Rudolph, Germany) connected to the MetaMax II. When the participants reached an oxygen consumption that was stable over 30 seconds, inclination (1–2% each step) or velocity (0.5–1 km·h^−1^) on the treadmill was increased depending on the appearance of and feedback from the participants until exhaustion. A maximal test was achieved with a respiratory quotient of 1.05 or higher or when the oxygen uptake did not increase >2.0 mL⋅kg^−1^⋅min^−1^ despite increased workload or before the participant disembarked the treadmill. Three participants (1 young athlete, 1 older athlete and 1 older non-athlete) did not attend the maximal exercise test, and valid VO_2max_ data was available for 17 participants.

### Statistical Analysis

Mean telomere length and oxygen uptake values were tested for normality and homogeneity of the variance. Shapiro-Wilk test provided strong evidence for normality (p = 0.80), and both Levene’s *F* test (p = 0.79) and Brown-Forsythe *F* test (p = 0.92) rejected the hypothesis that variances are unequal. We used independent t-test and analysis of variance to assess the difference in mean telomere length (T/S ratio) between groups for age and activity status. Pearson correlation analyses were used to assess the relationship between T/S ratio and maximal oxygen uptake. All analyses were one-tailed, *P*<0.05 was considered significant and analyses were conducted using SPSS, version 18 software.

## Results

Baseline characteristics of study participants are presented in Table 1. The mean age of young athletes was 24.4±0.6 years, and was not different than young non-athletes (23.6±2.7 years). Similarly, there was no difference in mean age of older participants (athletes, 69.2±2.9 years vs. non-athletes, 69.8±4.4 years). All participants reported to be free from known cardiovascular disease, no regular medications and no present or past history of smoking, and were not obese.

**Table 1:** Characteristics of study participants.

	Young		Old	
	Athlete	Non-athlete	Athlete	Non-athlete
Age, yr	24.4 (0.6)	23.6 (2.7)	69.2 (2.9)	69.8 (4.4)
Weight, kg	74.3 (4.4)	80.9 (13.6)	73.1 (7.8)	74.2 (4.6)
Height, cm	184.6 (3.9)	182.0 (5.5)	176.5 (3.1)	172.3 (5.1)
BMI, kg.cm^−2^	21.8 (0.7)	24.3 (3.4)	23.4 (2.9)	25.0 (1.1)
T/S ratio	1.47 (0.2)	1.33 (0.1)	1.12 (0.1)	0.92 (0.2)
VO_2max_, mL.kg^−1^.min^−1^	67.0 (5.3)	53.9 (5.5)	45.4 (6.7)	39.4 (5.6)

Values are mean (SD).

BMI, body mass index; VO_2max_, maximal oxygen uptake.

**Figure 1:**
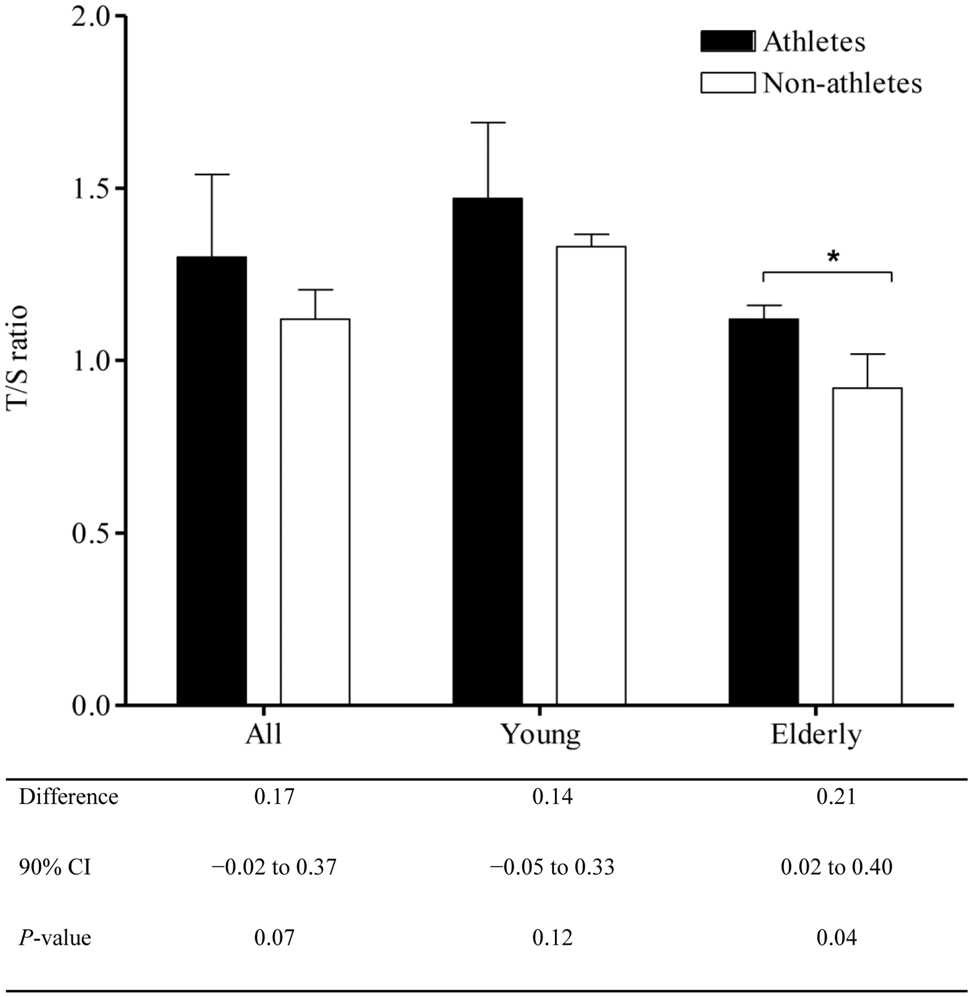
Telomere length expressed as T/S ratio among athletes and non-athletes, stratified by age. *P<0.05.

Figure 1 show that in older age group, endurance trained athletes had longer T/S ratio compared with medium activity levels (1.12±0.1 vs. 0.92±0.2, 90% CI; 0.02 to 0.40, p = 0.04). T/S ratio of young endurance trained athletes was not different from young non-athlete adults (1.47±0.2 vs. 1.33±0.1, 90% CI; −0.05 to 0.33, p = 0.12).

**Figure 2:**
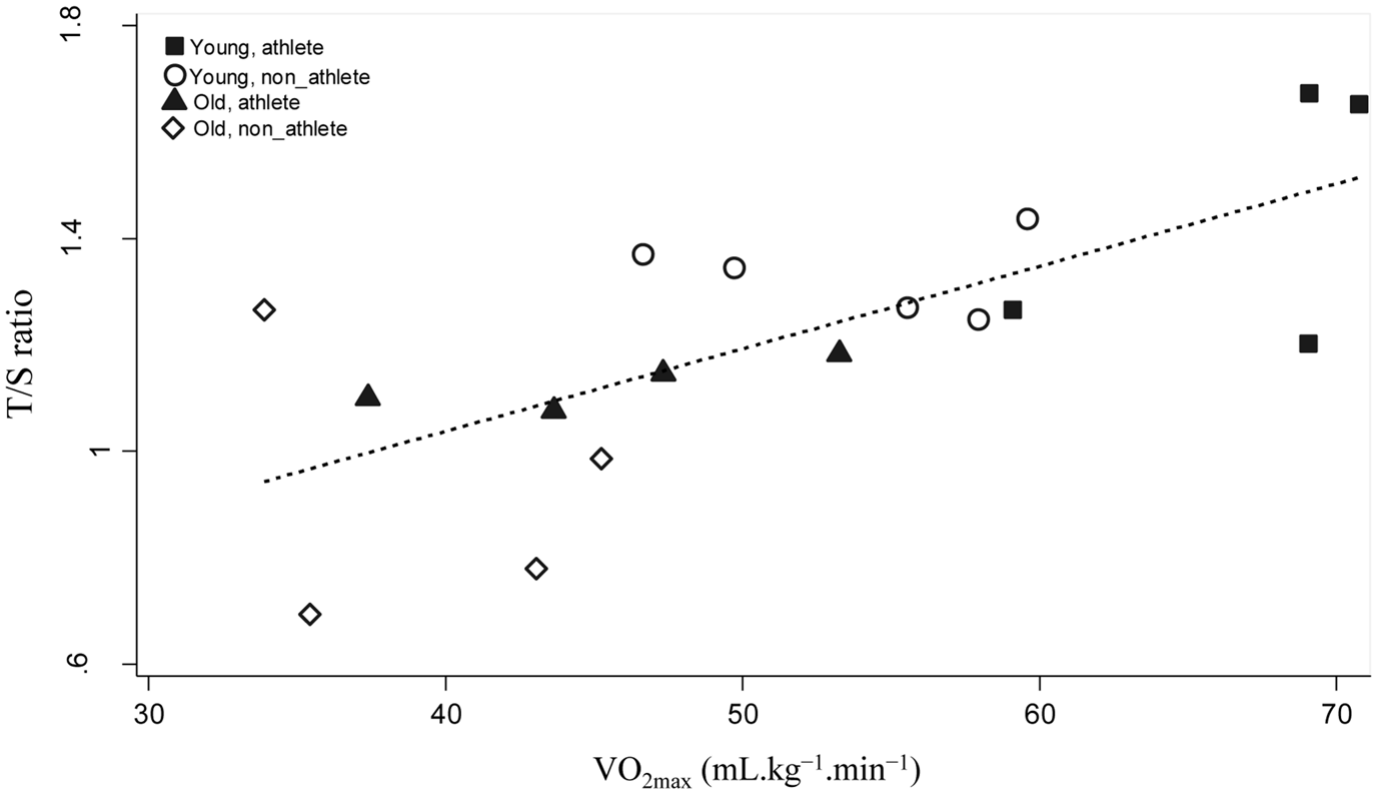
Telomere length (T/S ratio) and maximal oxygen uptake (VO_2max_) among athletes and non-athletes.

In young adults, the mean VO_2max_ was 67.0±5.3 mL·kg^−1^·min^−1^ for endurance athletes, and 53.9±5.5 mL·kg^−1^·min^−1^ among non-athletes. The analogous values of VO_2max_ were 45.4±6.7 and 39.4±5.6 mL·kg^−1^·min^−1^ among older participants. The aerobic fitness values of non-athlete groups corresponded closely with the mean values of oxygen uptake of general population residing in the same County [20]. Overall, there was a positive association between T/S ratio and VO_2max_ (r = 0.70, p = 0.001) as shown in Figure 2. Among endurance trained athletes, we found a strong correlation between VO_2max_ and T/S ratio (r = 0.78, p = 0.02). However, corresponding association among non-athlete participants was relatively weak (r = 0.58, p = 0.09).

## Discussion

We found that telomere length was better preserved in older endurance trained athletes than the same age group with a medium level of activity. In young people, we did not find an association between training status and T/S ratio. We also found a positive association between aerobic fitness and muscle telomere length in endurance exercise trained participants.

Previous studies have been inconsistent in showing the relative protective effect of exercise training on telomere length [9], [11]–[14], [21]–[23]. In a study by LaRocca et al. [13], older endurance trained athletes had longer telomere length than their sedentary peers. A recent study [11] suggested that even moderate amount of activity was associated with longer telomere lengths in women. Our findings are in line with these studies [12], [13], [24] in showing that long-term exercise training is associated with preserved telomere length in elderly people, but not with those reporting null associations [14], [15], [21].

In younger participants, we did not find an association between telomere length and physical exercise status. However, a recent study in healthy adolescents provided evidence for longer telomeres associated with greater amounts of activity in girls only [25]. In middle age participants, Cherkas et al found a dose-dependent association between leisure time physical activity and telomere length among women [12]. Interestingly, an inverted “U” curve association between increasing physical activity levels and telomere length is also reported [22], showing that low and high activity was associated with shorter telomeres and a moderate level of activity was associated with longer telomeres. These discrepant findings may be partly due to differences in physical activity measurements for intensity and duration of exercise and partly due to different populations and sample sizes.

The findings of our study should be interpreted with caution and being as preliminary. Unlike other studies where well-trained groups of participants were compared with more sedentary controls [9], [13], [23], or a short term training intervention was performed to assess the association with telomere length [15]; our study compared males with long-term physical activity at high intensity with healthy males at a medium activity level. Therefore, it is likely that non-athlete participants are healthier and more active than the sedentary general population, and this may have resulted in smaller difference of telomere length as observed in the present study. A non-significant association in young group might be due to reduced sample size, and a lesser exposure to physical activity in terms of activity years. Further, the difference in telomere length for older athletes and older people with medium activity levels may have clinical significance in relation to longevity; however, the design of the present study being cross-sectional in nature does not allow commenting about the causality of these results.

Various mechanisms [12], [22], [26] have been proposed for the telomeres shortening with ageing, although, little is known about their regulation in skeletal muscles [26], [27]. The observed preservation of telomeres in old healthy endurance athletes suggests some *in vivo* regulatory mechanisms including but not limited to improved mitochondrial function, oxidative stress induced up regulation of telomerase, and other life stress situations and diseases [23], [26], [27]. Furthermore, several measures of physical fitness decrease with age, and so seems to be the case for telomere length as well. Whether shortening of telomeres is direct effect of the physical ageing process, or rather a co-existing measure for biological age remains unresolved.

Our results also suggest a positive association between maximal oxygen uptake and telomere length, and provide further support to the hypothesis [13] that long-term exercise, higher aerobic fitness and longer telomeres all are part of same phenotype expressed in some older adults.

Except for exercise training status, the groups of participants in present study were comparable based on age, non-smoking status, non-obese and without any prevalent cardiovascular disease or without on medications. At the start of study, we did not have information about the effect size and therefore could not perform sample size estimation and power calculation as an *a priori*. However, we report difference in mean telomere length with 90% confidence interval among different groups because *a priori* power estimations are immaterial at the end of study, and it is the size of effect estimates and the width of the confidence interval that is important [28]. Nonetheless, small sample size is a limitation of the present study together with only male participants. Furthermore, the variability in telomere length has been observed in skeletal muscles and other tissues [27], suggesting that telomere length can be shortened with ageing in skeletal muscles and not in blood or liver within the same individual [29]. However, a recent study has shown that muscle telomeres are positively correlated with the leucocytes telomeres, and both can be used as proxy to each other [30]. The specific tissue to measure telomere length should be decided depending on study design keeping in accordance with Helsinki Declaration. In the present study, blood leucocytes would have been a convenient approach to assess the telomere length; however, muscle biopsies from our study participants have been used in other studies [31], [32] and extra blood samples would have been unnecessary. For the association of telomere length and exercise training, we cannot exclude the possibility of residual confounding because of unknown or unmeasured factors, such as vitamin supplementation or antioxidant intake and oxidative stress measurements, for which the information was not available in the present study.

Our results suggest that endurance exercise training may regulate the telomeres in old age, and results in slowing of ageing process by maintaining telomere length. The positive association of VO_2max_ and telomere length underscores the importance of aerobic fitness for healthy ageing. Large-scale prospective and longitudinal studies are warranted to assess the role of long-term exercise and VO_2max_ on telomere length with ageing.
